# Eco-Friendly and Highly Efficient Enzyme-Based Wool Shrinkproofing Finishing by Multiple Padding Techniques

**DOI:** 10.3390/polym10111213

**Published:** 2018-10-31

**Authors:** Le Wang, Jinbo Yao, Jiarong Niu, Jianyong Liu, Bo Li, Mao Feng

**Affiliations:** 1 School of Textiles, Tianjin Polytechnic University, Tianjin 300387, China; wangletianjin@163.com (L.W.); niujiarong@tjpu.edu.cn (J.N.); jianyong1964@126.com (J.L.); 1988li01bo22@163.com (B.L.); 52x@msn.com (M.F.); 2 School of Chemistry and Chemical Engineering, Wuhan Textile University, Wuhan 430073, China

**Keywords:** enzyme, wool, activator, shrinkproofing, padding

## Abstract

Wool fibers usually need shrinkproofing finishing. The enzyme process is an eco-friendly technology but the traditional exhaustion treatment usually takes excessive time. This study developed a novel multiple padding shrinkproofing process of wool with Savinase 16L and an organic phosphine compound {[HO(CH_2_)_n_]_3_P, n ∈ (1, 10)}. SEM and XPS analyses were employed to compare the wool treated respectively by exhaustion and by padding to reveal the effect of multiple padding. The results showed that treated wool fiber achieved the requirement of machine-washable (area shrinkage less than 8% according to standard TM 31 5 × 5A) in 2.5 min by the padding process. The padding process can control the adsorbance of enzyme on wool, which makes treatment more uniform and avoids strong damage of the wool. Also, the removal efficiency of the disulfide bond was about 15 times as much as in the exhaustion treatment in 2.5 min. The average catalytic rate of the padding process was 14 times faster than the exhaustion process, and the process time (2.5 min) decreased by 32.5 min compared with the exhaustion process (35 min). Multiple padding techniques can achieve continuous production and replace the environmentally harmful chlorination process. Our results provide the underlying insights needed to guide the research of the enzyme process application.

## 1. Introduction

Wool is a type of high-grade natural textile material. It has many features such as soft to the hand, elasticity, etc. However, wool fibers have cuticles, which make wool fabrics unstable and easy to shrink and deform in the washing process. Therefore, in order to improve the product’s dimensional stability, the scales on the surface of the wool fiber need to be processed to reduce its directional friction effect (D.F.E.) and to be machine-washable [1].

The Chlorine-Hercosett treatment that combines mid-chlorination pretreatment with cationic polymer deposition on wool fiber surface is most widely used in conventional wool shrinkproofing finishing. The chlorination process causes fiber yellowing, and the use of resin results in the fiber being stiffer to handle. What is more, during the mid-chlorination pretreatment, a large amount of chlorine gas is released into the air, which threatens the health of the operator and is seriously detrimental to the atmosphere. Adsorbable organic halogens (AOX) are generated by reaction of chlorine with wool amino acid residues in the process of chlorination [2]. There is a high concentration of AOX in the waste liquid. AOX not only pollutes the environment but also causes serious damage to humans [3]. Chlorine-Hercosett technology is unsustainable and does not meet the requirements of eco-textiles processing. However, there is still no effective treatment method that can replace chlorine treatment in wool fibers shrinkproofing finishing. Therefore, when the eco-label (2002/371/EC) was established by the European Community for textile products, they were forced to make the following decision: “*Halogenated shrink-resistant substances or preparations shall only be applied to wool slivers*”. It is urgent to study a cleaner, efficient, and industrialized wool shrinkproofing technology.

At present, many sustainable technologies for eco-friendly processes of wool shrinkproofing methods have been brought forward under the concept of environmental protection, including enzyme treatment [4], plasma processing [5], ozone gas processing [6], and processing with nano materials and so on [7]. The enzyme method is not only eco-friendly but also has mild conditions [8]. From the point of view of cleaner industrial application, enzyme treatment is the most likely to replace the chlorination treatment of these research methods. More and more researchers have focused on the shrinkproofing treatment with enzymes [9]. Part of the scales of wool can be removed by enzyme treatment through the hydrolysis reaction of enzymes on the peptide bonds of wool fibers [10]. Shrinkproofing finishing with enzyme is a kind of environmentally friendly method which has many extra advantages such as easy operation, saving energy, and so on [11].

However, there are two main restricting factors for the cleaner industrial development of shrinkproofing finishing with enzyme. On the one hand, the reaction of enzyme with wool takes a long time to process and it is difficult to achieve the requirement of machine washable of the treated wool only through enzyme treatment [12] because there are a lot of hard keratins that have a high content of disulfide bonds in the cuticles of the wool fiber [13]. Disulfide bonds inhibit the reaction of the enzyme macromolecules with the wool scales. Therefore, a combination of pretreatment and enzyme exhaustion treatment is widely used in wool shrinkproofing finishing [14], such as KMnO_4_ pretreatment, H_2_O_2_ pretreatment, etc. [15], which is time-consuming and uncontrollable [16]. On the other hand, the mechanical properties of wool fibers treated by enzyme are usually significantly affected by damage [17]. Enzyme molecules diffuse to the inside of the wool fibers during the extensive time of enzyme treatment and destroy the wool fiber cortex [18], causing irreversible damage to the wool fibers. Methods of transglutaminase treatment and graft modification of enzyme and polymer deposition have been used to improve the wool tensile strength after treatment [19]. Transglutaminase can catalyze the cross-linking reaction between protein molecules and improve the mechanical properties of the wool fiber. Transglutaminase treatment was reported to be used in improving the tensile strength of damaged wool fibers [20] but the increase of fiber tensile strength is limited through cross-linking of proteins. In order to inhibit the enzyme molecular diffusion to the cortex and reduce the mechanical damage of wool in extended time finishing with enzyme, the molecular weight of the enzyme can be increased by graft modification [21]. Jinsong Shen [22] proposed modifications to the proteases so that the reaction between enzyme molecules and wool was restricted to the cuticle scales of the fibers. However, the enzyme activity and the homogeneity of the treatment are affected by the graft modification [23]. In addition, the method of polymer deposition with organics or resin has also been suggested to improve the tensile strength of the treated wool fiber [7]. However, the feeling of the wool becomes rough by the polymer deposition process. Consequently, the enzymatic treatment of wool shrinkproofing can hardly be industrialized [24].

In order to realize the industrialization of cleaner and environment-friendly shrinkproofing finishing with enzyme, it is extremely necessary to improve the efficiency of action and reduce the time of enzyme treatment. The enzyme treatment must be completed before the enzyme diffuses into the cortex and the cell membrane complex of the wool fiber. In this way, the reaction between the enzyme and the wool fiber is controlled only on the surface of the fiber. To achieve rapid enzyme shrinkproofing finishing, the rate of migration of the enzyme on the surface of the wool must be accelerated, so that enzyme acts evenly on the surface of the wool fiber in a short time to avoid its irreversible damage to the wool at the adsorption position. Therefore, multiple padding treatments can be used to achieve rapid enzyme shrinkproofing finishing by improving the migration efficiency of the enzyme on the wool surface during the process.

In this work, the wool top was treated with Savinase 16L and activator in one bath in shrinkproofing finishing by using multiple padding techniques. The activator does not only maintain enzyme activity, but also can break down disulfide bonds in the wool scales [25]. The adsorption and reaction properties of enzymes on wool were investigated. The mechanism of rapid and highly efficient shrinkproofing finishing by multiple padding was explored. All these investigations were expected to guide future industrial application of eco-friendly and high efficient enzyme-based wool shrinkproofing technology.

## 2. Materials and Methods 

### 2.1. Materials

The wool tops (70s Australian wool) were kindly provided by Changshu Xinguang Wool Top Treatment Co. Ltd. (Changshu, China). The Savinase 16L (activity 400,000 u/mL) was supplied by Novozymes. {[HO(CH_2_)_n_]_3_P, n ∈ (1, 10)} was named as “activator” in this work, which was obtained from Tianjin Lvyuan Tianmei Science and Technology Co. Ltd., Tianjin, China. Chemical reagent including penetrant (fatty alcohol–polyoxyethylene ether) and cationic silicone softener were supplied by Binhai Dongfang Science and Technology Co. Ltd., Hebei, China.

### 2.2. Enzyme Treatment 

#### 2.2.1. Exhaustion Process

A scoured wool top was wetted with 1 g/L penetrant solution at 50 °C for 30 s having a bath ratio of 100:1 (liquor to wool). After which the wool sample was treated with shrinkproofing treatment solution that was mainly composed of 1 mL/L Savinase 16L and 2 mL/L activator at 50 °C and pH 8.0 for 2.5 min with a liquor ratio of 100:1, followed by rinsing with water at 45 °C for 30 s and enzyme inactivated with water at 80 °C for 30 s. Finally, the sample was softened in 1 mL/L softener solution at 45 °C and then dried at 80 °C in an oven (Shanghai Kord Laboratory Equipment Co. Ltd., Shanghai, China).

#### 2.2.2. Multiple Padding Process

Multiple padding treatments for scoured wool top were carried out with a padder (Laizhou Yuanmao Instrument Co. Ltd., PO-B, Laizhou, China). The speed of padder was 6 m/min, and the liquid pick-up was 80% ± 5%. Scoured wool top was wetted with 1 g/L penetrant solution at 50 °C for 30 s followed by squeezing with the padder. Then the wool sample was soaked for 30 s with shrinkproofing treatment solution that was mainly composed of 1mL/L Savinase 16L and 2mL/L activator, at 50 °C and pH 8.0 followed by squeezing with the padder. The shrinkproofing treatment operation was repeated 5 times and the frequency of padding was 30 s each time (total time 2.5 min), followed by rinsing with water at 45 °C for 30 s and enzyme inactivated with water at 80 °C for 30 s. Finally, the sample was softened in 1 mL/L softener solution at 45 °C and then dried at 80 °C in an oven.

### 2.3. Plant Treatment of Wool with the Chlorine-Hercosett Process and Multiple Padding Enzyme Process Respectively

**Figure 1:**
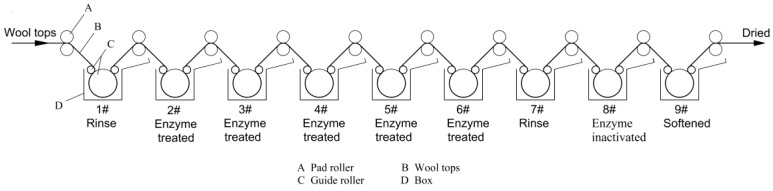
Schematic of multiple padding enzyme treatment in the plant.

Scaling-up for the industrialization process was carried out at Changshu Xinguang Wool Top Treatment Co. Ltd. Conventional Chlorine-Hercosett is a continuous production. The scoured wool top was wetted with 1 g/L penetrant solution at 50 °C for 30 s, after which the wool was chlorinated with 0.15 g/L NaClO at pH 1.5 (adjusted with H_2_SO_4_) at 25 °C for 2 min. After chlorination, the sample was treated with 5.0 g/L Na_2_SO_3_ at pH 9.0 (adjusted with NaHCO_3_) at 25 °C for 2 min to remove residual traces of chlorine from the wool fibers, followed by rinsing with water at 45 °C two times. Then the sample was treated with 5 g/L hercosett polymer (resin) pH 7.8 (adjusted with NaHCO_3_) at 25 °C for 2 min. Finally, the sample was softened in 1 mL/L softener solution at 45 °C and then dried at 80 °C in an oven. The multiple padding enzyme process was performed as Section 2.2.2. It is also a continuous process. The schematic is shown in Figure 1. The volume of the treatment box is 500 L. The wool top feeding speed is 6 m/min. The length of wool top is 3 m between two pad rollers.

### 2.4. Measurement

#### 2.4.1. Felting Shrinkage

The felting shrinkage of wool fibers was characterized according to the International Wool Textile Organization (IWTO) test method TM 31. Wool fibers were woven into wool fabrics according to standard. The wool fabrics were subjected to multiple wash and tumble dry cycles. The size of a knitted sample was 300 × 400 mm^2^. Wool fabrics were subjected to 7A program for relaxation shrinkage. Then, shrinkage tests were conducted with 5A program 5 times. The test results are expressed as the percentage of felting shrinkage (area shrinkage) after relaxation. Note: The way of drying was flat to dry.

#### 2.4.2. Directional Friction Effect

Directional friction effect was measured by a fiber friction coefficient tester (Changzhou First Textile Machinery Factory, Y151, Changzhou, China) under standard atmosphere [26]. The sample must be stored in a standard atmosphere (temperature 20 ± 2 °C, relative humidity 65% ± 2%) for 48 h before testing. The fiber pretension was 100 mg and the testing speed was set at 30 rpm. The fiber frictional force was measured against a metal rod of diameter 8 mm and the mean value was calculated after testing 50 fibers. The friction coefficient and the directional friction effect were obtained according to Equations (1) and (2):

*μ* = In [f/(f − *m*)]/π (1)

*D.F.E.* (%) = (*μ_a_* − *μ_s_*) * 100/(*μ_a_* + *μ_s_*) (2)

where, f represents tension of standard clamp weight (fixed value 100 mg) and *m* is the value of the fiber friction coefficient tester (mg). *μ* stands for friction coefficient. *μ_a_* is the coefficient of friction against the direction of scales and *μ_s_* is the coefficient of friction along with the direction of scales. *D.F.E.* means directional friction effect.

#### 2.4.3. Mechanical Property

The tensile strength of wool fibers was measured by a fabric tensile strength tester (Laizhou Yuanmao Instrument Co., Ltd., YM-06A, Laizhou, Shandong, China). For tensile properties, 300 single fibers were tested in a mini tensile machine applied on jute fiber with a pretension of 0.1 cN, gauge length of 10 mm, and clamp speed of 10 mm/min. The test must be carried out under standard atmosphere. The test sample must be stored in the standard atmosphere for 48 h before testing. Tensile strength retention rate and tenacity retention rate were respectively calculated according to Equations (3) and (4):

Tensile strength retention rate (%) = (*F’*/*F*) × 100 (3)

Tenacity retention rate (%) = (*δ’*/*δ*) × 100 (4)

where *F* is the mean value of the tensile strength of control. *F’* is the mean value of the tensile strength of treated sample. *δ’* and *δ* are respectively the mean values of the tenacity of the treated sample and control.

#### 2.4.4. Fineness

The fineness of the fiber samples was measured by a fineness tester (Apex Measurement Technology Co. Ltd., OFDA 4000, Shanghai, China) according to IWTO TM 62. The test was carried out under standard atmosphere. The samples were stored in the standard atmosphere for 48 h before testing.

#### 2.4.5. Surface Morphology

The surface morphology of the control and treated fibers was studied using a cold field emission scanning electron microscope (SEM S4700, Hitachi, Tokyo, Japan). Samples were sputtered with gold under vacuum prior to observation. The SEM images were taken at an accelerating voltage of 10.0 kV.

#### 2.4.6. Enzyme Adsorption

Enzyme activity in solution was determined by China criteria of GB/T 23527-2009 protease preparations appendix B folin’s method, and the adsorption amount of enzyme on wool fiber surface was calculated with Equation (5):

(5) $$Q=(U-U^{\prime })\times V/M$$

where *U* denotes the initial enzyme activity in the treatment solution before treatment (u/mL). *U*′ is the enzyme activity in solution after treatment of wool (u/mL). *V* is the volume of the treatment liquid (mL). *M* is the mass of the wool fiber sample (g) and *Q* is the quantity of the amount of enzyme adsorption (u/g wool).

#### 2.4.7. Amino Acid Content

The amino acid content of wool samples was analyzed by an amino acid analyzer (membraPure Co. Ltd., A300, Hennigsdorf, Germany). Samples were not softened before testing. When the samples were analyzed by the amino acid analyzer, the Na salt system was selected, the column temperature was set to 40 °C, the reactor temperature was set to 115 °C, the flow rate of ninhydrine was 125 yards per minute and the buffer was 250 yards per minute. The pressure of the eluent was 60–70 bar and the regenerated liquid was 10–20 bar.

#### 2.4.8. Weight Loss

Wool fibers were conditioned at 100 °C for 4 h, desiccated and weighed until constant weight (considered as differences between successive weights inferior to 1 mg). The measurements were carried out in duplicate.

#### 2.4.9. X-ray Photoelectron Spectroscopy (XPS)

XPS spectra were recorded using a Thermo Scientific K-Alpha XPS instrument fitted with an Al K Alpha radiation (1486.6 eV) in the following conditions: spot size = 400 μm, pass energy = 50 eV and step size = 0.1. The X-ray anode was run at 250 Wand. The high voltage was kept at 14.0 kV.

#### 2.4.10. Colorimetric Measurements

The CIE LAB colorimetric system was used for determination of color difference of control and treated samples by using a Datacolor 800. Light source was CIE Illuminant D65. L* (lightness and darkness), a* (redness and greenness), b* (yellowness and blueness), CIE whiteness could be measured at the same time.

## 3. Result and Discussion

### 3.1. Effect of Different Enzyme Treatment Methods on Wool Fiber Properties

#### 3.1.1. Shrinkproofing Performance and Surface Morphology

**Figure 2:**
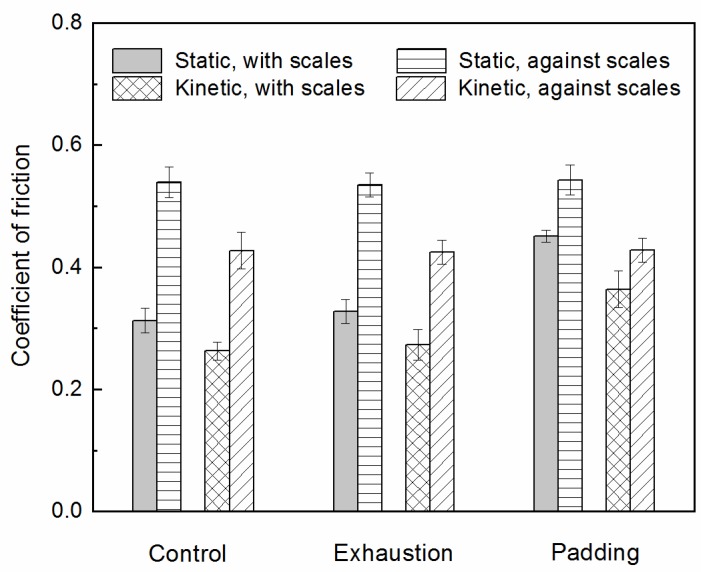
Effect of different treatments on friction coefficient.

The shrinkage of wool textiles in the presence of water and mechanical agitation is mainly due to the directional friction effect (D.F.E.) induced by the surface scales [27]. Modification of the scale structure of the wool fiber cuticle to decrease the D.F.E. can make wool fabrics withstand repeated washing without shrinkage and felting [26]. Wool top was treated by the exhaustion and padding treatments respectively. Table 1 shows the percentage of felting shrinkage and the D.F.E. Figure 2. shows the coefficient of friction before and after different treatment.

The area shrinkage ratio was 49.2% after the exhaustion process, which did not meet the requirement of the machine washable (TM 31 5 × 5A shrinkage ratio less than 8%). In contrast, the shrinkage rate was 2.4% after the multiple padding process that met the requirements of the machine washable. The importance of mechanical padding on the wool shrinkage treatment with enzyme can be seen.

The static and kinetic D.F.E. of wool samples were reduced slightly after the exhaustion treatment and the against-scales friction coefficient was almost unchanged. After the multiple padding process, the static and kinetic D.F.E. were obviously reduced. The static D.F.E. was reduced by 65.1% and the kinetic D.F.E. reduced by 66.0%. Compared with control sample, the against-scales friction coefficient was almost constant and the friction coefficient of with scales was obviously improved after the multiple padding process (Figure 2). The wool fiber surface became rough when the Savinase 16L and activator reacted with the scales of the wool fiber. Therefore, the coefficient friction of with scales was increased. The wool treated with the enzyme and activator met the requirement of rapid shrinkproofing by reducing the D.F.E. through increasing the coefficient friction of with scales. This was also confirmed by electron micrographs of the fiber surface.

**Figure 3:**
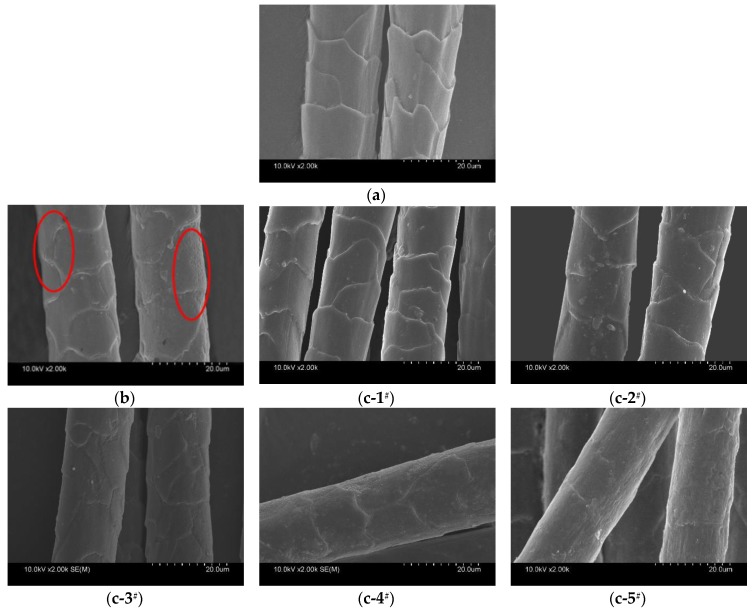
SEM images of wool fibers (**a**) control (**b**) exhaustion treated with 1 mL/L Savinase 16L and 2 mL/L activator, at 50 °C, pH 8.0, bath ratio 100:1 for 2.5min and (**c-1^#^**)–(**c-5^#^**) Padding treated with 1 mL/L Savinase 16L and 2 mL/L activator, at 50 °C, pH 8.0, (**c-1^#^**) Padded 1 time,…, (**c-5^#^**) Padded 5 times.

The morphology of the fiber surface after exhaustion treatment is shown in Figure 3b. Compared with the control sample (Figure 3a), it could be observed that part of the fiber surface scales remained substantially complete, while part of the wool fiber scales was removed or slimmed down after exhaustion processing. The reaction was not uniform and a large number of holes appeared on the fiber surface. The holes contributed to the increase of the with scales friction coefficient (Figure 2). It was suggested that Savinase 16L and activator can be used to treat wool fiber and the wool fiber scales can be modified or even stripped in a short time. However, the effect of exhaustion treatment was not uniform. It was mainly caused by the fact that the enzyme had a high molecular weight (20–30 KD). The enzyme molecule has difficult in migrating when it is adsorbed on the surface of wool fibers [18]. Adsorbed enzyme would continuously act on the inside of the wool at the binding site of the wool fiber, destroying the fibrous cortex and forming a hole on the surface of the fiber scales [28]. Consequently, it was not uniform for the reaction of enzyme and wool with the exhaustion treatment. It is hard to make the enzyme act only on the surface of the wool fiber.

**Figure 4:**
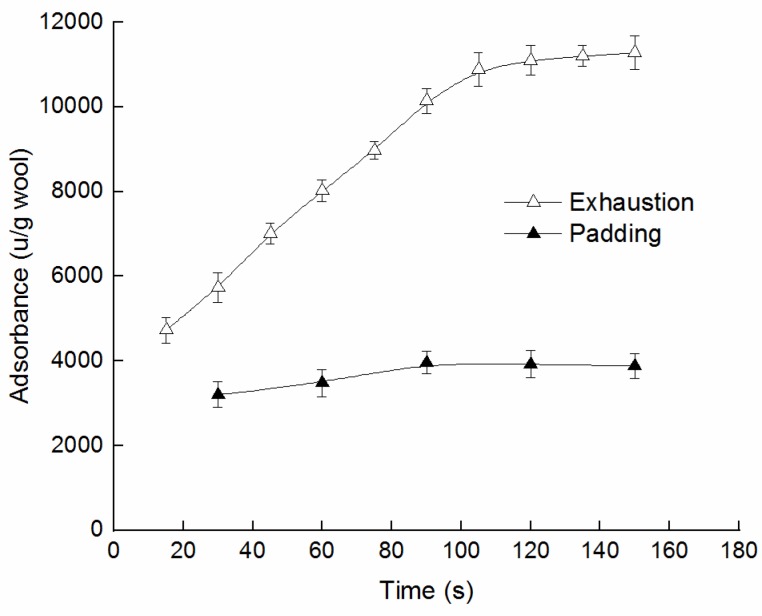
The adsorption of Savinase 16L with time in different treatments.

**Figure 5:**
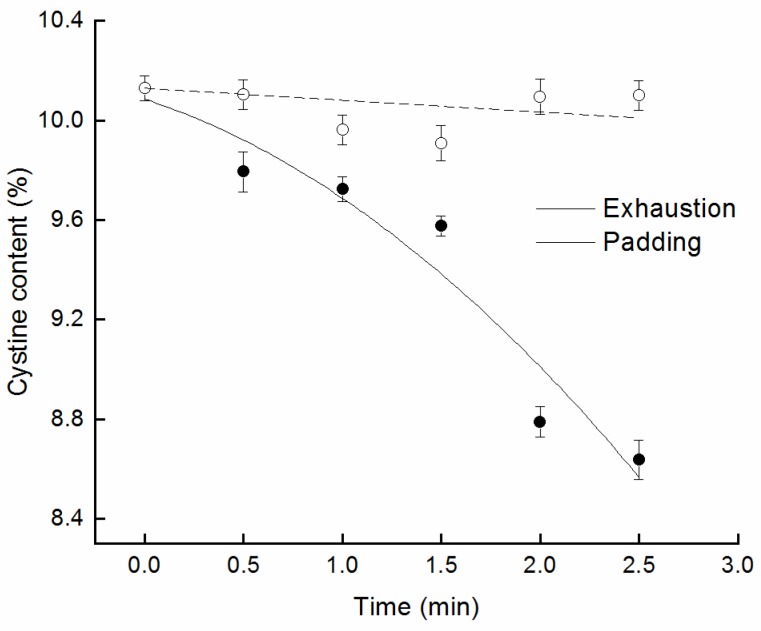
Effects of different processes on cysteine content.

Figure 3(c-1^#^)–(c-5^#^) shows the change of scale during the padding process. It can be seen that wool scales were gradually stripped with the times of padding. The surface morphology after multiple padding treatments for 2.5 min is shown in Figure 3(c-5^#^). It can be seen that the scales were stripped. Holes did not appear on the surface of the wool fiber treated by multiple padding. The surface of the wool fiber was relatively rough after treatment. The friction coefficient of with scales obviously increased and the friction coefficient of against-scales was almost constant (Table 1) while the multiple padding technologies effect was uniform. The enzyme was forced to be adsorbed and desorbed on the surface of the wool fiber by multiple padding, which also promoted the migration of enzyme macromolecules on the wool fiber surface. Therefore, multiple padding treatments are uniform and avoid the diffusion of enzymes into the cortex. The internal structure of the wool fiber is protected by the multiple padding process.

#### 3.1.2. Fineness and Mechanical Properties

Wool was treated by the exhaustion and padding process respectively. The fineness and mechanical properties of the control and treated samples are shown in Table 2. It can be seen that the fineness and strength value of the wool fibers gradually decrease with the increase of padding times when the wool sample is treated by multiple padding technology. It also shows that the fineness of the fibers treated by exhaustion was only reduced by 0.1 μm, and the fineness of the fiber treated by padding was reduced by 0.6 μm after padding, treated five times. Although the treating time and concentration of chemicals were the same, the averaged tensile strength and tenacity of the wool fiber were less damaged after the exhaustion treatment, the fineness was almost unchanged. However, the fineness was reduced to 18.90 μm and wool scales were partially removed by the multiple padding treatment. This phenomenon indicates that multiple padding treatments have a higher efficiency of stripping wool scales than the exhaustion process. The removal of the scales will inevitably cause damage to the mechanical properties [18]. However, the treatment time is only 2.5 min by Savinase 16L and activator through the multiple padding process so that the excessive damage can be avoided. Then the tenacity retention rate of the wool fiber was 92.6% after multiple padding treatments, which was in an acceptable range.

### 3.2. Effect of Different Enzyme Treatment Methods on Adsorption Performance of Enzyme

The reaction of the enzyme with the substrate is a contact reaction. In other words, the enzyme must firstly adsorb and combine with the wool fiber, and then the wool fiber peptide bond can be hydrolyzed effectively. Therefore, it is significant to control the enzyme adsorption only at the cuticle layers. Amandeep Kaur [17] controlled the adsorption of bromelain at the outer surface of wool in the presence of salt. In this research, it was found that multiple padding can prevent enzyme diffusion into inside the fiber.

The Savinase 16L adsorbances of the exhaustion method and padding method are plotted in Figure 4. It can be seen that the enzyme adsorbance increases with the increase of time in the exhaustion process. The enzyme adsorbance and exhaustion time were almost proportional at the initial stage. Adsorption equilibrium was reached when the exhaustion time reached 120 s. There was a balance between the rates of enzyme absorption and desorption [29]. The enzyme adsorbance was about 11,000 u/g. With the prolongation of processing time, the increase of adsorbance was not obvious by the padding treatment. The adsorbance of enzyme was 3200 u/g after padding for the first time. The enzyme adsorbance of the wool fiber almost did no longer change during the following paddings.

Although enzyme adsorbance was much bigger for wool by the exhaustion treatment, the cuticle was almost unstripped without the mechanical padding action. It may be due to the fact that the enzyme molecular weight is large which makes it difficult for the enzyme to migrate [18]. The enzyme molecules that had been adsorbed on wool no longer migrated and continued to react onto the wool fibers at the initial adsorption position. As a result, ‘holes’ were formed (Figure 3b). It was difficult to peel off the wool scales in a short time, so the fineness changed little (Table 2). 

For the padding treatment, with the wool that had treatment liquid passed through the roll, the adsorbed enzyme molecules were forced to transfer by the flow between fibers. The squeezing promoted the mobility of the enzyme macromolecule and the rates of enzyme absorption and desorption. Moreover, mechanical pressure makes the wool fibers rub against each other. The tangential force also made the wool scales that had been partially hydrolyzed by enzymes fall off. At the same time, the adsorbed enzyme molecules on the scales also separated from the wool fibers and then new scale tissue that had not been in contact with treatment solution was exposed. In the next padding round, the enzyme and activator reacted further with the newly exposed portion [30], so that multiple padding could promote the stripping of the wool scales. It can be seen that the adsorbance of the enzyme was nearly unchanged during the padding process, but the rates of adsorption and desorption were accelerated. Consequently, the wool scales were uniformly treated. The scales rapidly fell off layer by layer and the diameter of the wool also became slender.

### 3.3. Effect of Different Enzyme Treatment Methods on Reaction Efficiency

There are a lot of disulfide bonds in wool scales. This can prevent the catalytic degradation of wool scales by enzyme [18]. Figure 5 shows the change of cysteine content of the wool fiber. It is obviously related to the change of the cuticle enriched disulfide bond. It can be seen from Figure 5 that the cysteine content of the wool fiber changed with processing time when wool fiber was soaked in the treatment solution of Savinase 16L and activator. Meanwhile, the cysteine content of wool fiber dramatically decreased in the multiple padding treatments and the removal efficiency of cysteine was about 16 times as much as in the exhaustion treatment in 2.5 min. Contact and reaction between the activator molecule and the disulfide bond were promoted by the padding process. Then a large number of disulfide bonds in the scales were broken down. This was beneficial to the reaction of macromolecular enzymes with wool fiber scales [1].

**Figure 6:**
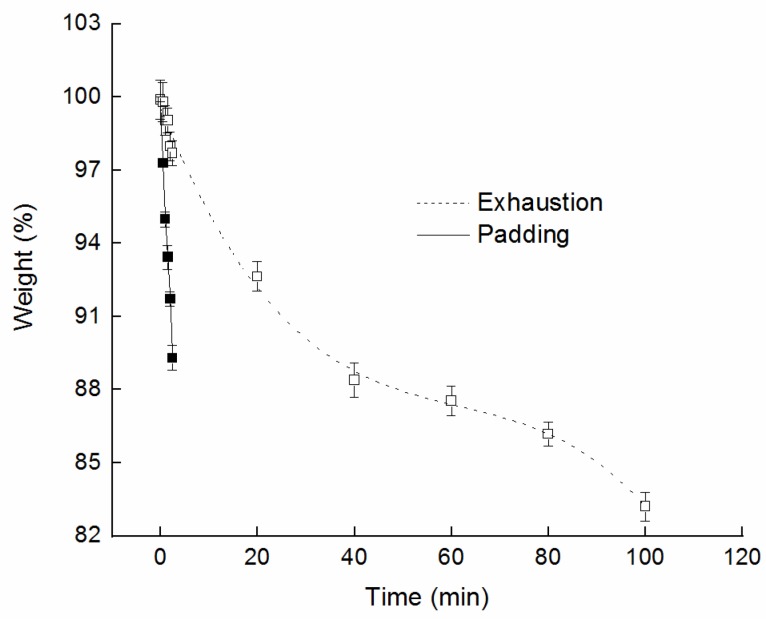
Effects of different processes on weight loss.

The changes of wool weight are shown in Figure 6. It was evident that the reaction rate of the padding treatment was more rapid than the exhaustion treatment. The weight loss was 10.5% in 2.5 min after multiple padding treatments while it took 35 min for the exhaustion process to achieve the same weight loss. The reaction rate of the padding process was 14 times faster than the exhaustion method. There are two main reasons for the high reaction rate of the padding process. First of all, high-speed flow was produced between wool fibers at the moment of mechanical squeeze. The squeezing prompted the migration of enzyme molecules. Meanwhile the rate of collisions was raised between enzyme and wool fiber, so that the rate of reaction was improved while the holes that caused enzyme combined with wool fiber to act continuously at a point was avoided. The reaction product of the enzyme and the wool fiber could be extruded through the squeezing effect of the roller. The concentration of reaction product decreased rapidly on the wool fiber surface, and the effect of product inhibition was also reduced. Secondly, the epicuticle which reacted on the wool surface partly swelled or fell off during the extrusion process. The exocuticle and endocuticle become exposed. This increases the accessibility of enzyme and promotes the further reaction of enzyme onto the wool scales [30]. Therefore, the content of amino acids was obviously reduced.

### 3.4. XPS Characterization of Wool Fibers after Enzyme Treatment

The investigation of surface elemental composition of wool samples before and after treatment was carried out using XPS, which is sensitive [31]. The relative atomic concentrations of the fiber surface after treatment is summarized in Table 3.

The surface atomic composition of C and N element was increased after exhaustion treatment. The result indicated that a large number of peptide bonds was hydrolyzed by enzyme, a great deal of –NH_2_ was exposed. The atomic composition of O significantly increased after the padding treatment, which may be related to the exposure of the endocuticle while part of the disulfide bonds broke down and oxidized further. The atomic composition of S decreased obviously after both processes respectively. although more significantly through the padding process.

**Figure 7:**
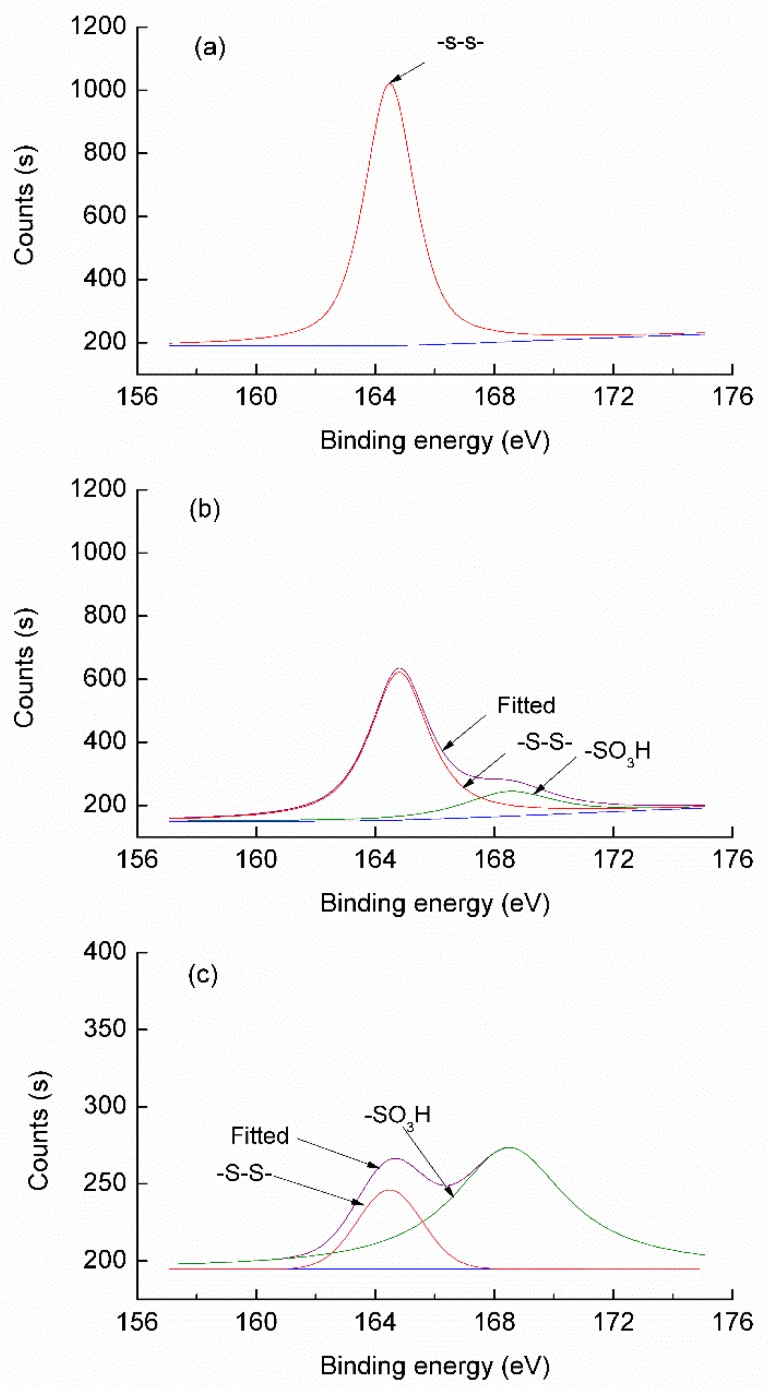
Element S 2p spectra of wool fibers XPS (**a**) control (**b**) exhaustion treated (**c**) padding treatment.

The S 2p spectrum was deconvolved with XPS PEAK software as shown in Figure 7. Table 4 summarizes the relative amounts of differently bonded peaks at 164.48 eV, 168.5 eV corresponding to –S–S– (S^2+^) and –SO_3_H (S^6+^) bonds respectively [32]. The component of the –S–S– (S^2+^) in the 164.48 eV decreased after the exhaustion or padding treatment. This shows clearly that the treatment solution of Savinase 16L and activator can destroy the disulfide bonds of wool. After the different process, a newly separated peak at 168.5 eV was observed in comparison with control sample. The peak at 168.5 eV corresponds to –SO_3_H explained by the fact that –SO_3_H was partially generated after processing with Savinase 16L and activator, which was derived from the oxidation of the partially opened disulfide bonds [33]. Compared with the exhaustion process, the component of the –S–S– (S^2+^) at 164.48 eV decreased significantly and the oxygen containing group –SO_3_H increased noticeably after padding treatment. The results indicate that more disulfide bonds in the cuticle are cleaved during multiple padding, resulting the formation of –SO_3_H.

The destruction of the disulfide bonds was accelerated on the surface of the wool by the multiple padding process. As a result, the accessibility of enzyme was increased, which promoted the efficiency of the enzyme. The scales of the wool were quickly hydrolyzed and stripped off in a remarkable fashion by the padding process. Therefore, the shrink performance of treated wool can reach the machine-washable state in a short time.

### 3.5. Performance Analysis of Chlorine-Hercosett Treated and Multiple Padding Enzyme Treated Wool

**Figure 8:**
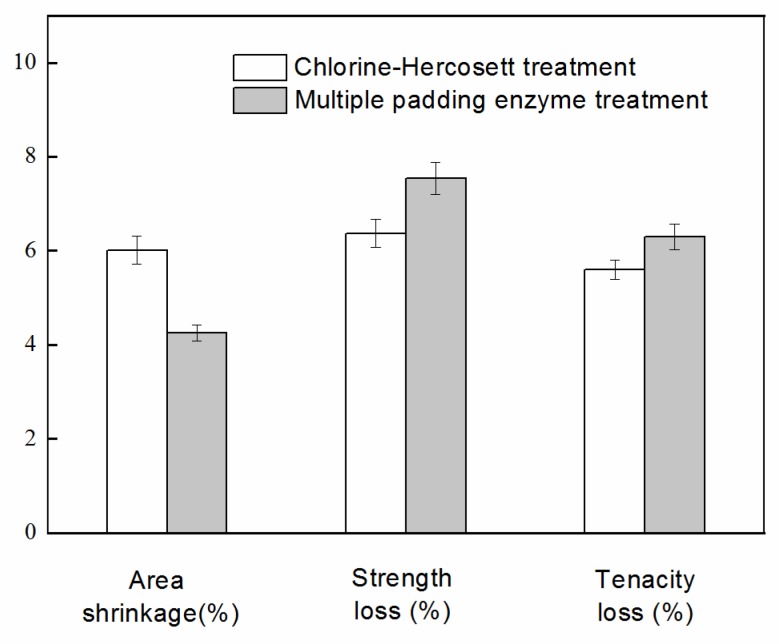
Shrinkage and mechanical properties after treatment.

Comparative analysis of the properties of the Chlorine-Hercosett treated and multiple padding enzyme treated wool at the plant is shown in Figure 8. Both of the continuous processes could meet machine washable requirements (area shrinkage <8%). The shrinkage performance of enzyme treatment (4.3%) is better than that of the Chlorine-Hercosett treatment (6.0%). Less strength loss and tenacity loss after the Chlorine-Hercosett process may be due to hercosett polymer deposition [34]. Color change after Chlorine-Hercosett treatment and multiple padding enzyme treatment is shown in Table 5. Compared with the Chlorine-Hercosett process, the whiteness of the wool fiber was obviously improved after enzyme treatment. In contrast to the control sample, the color of enzyme treated wool shifted towards bluer (∆b* < 0) while Chlorine-Hercosett treated shifted towards yellower (∆b* > 0) [35]. In other words, Chlorine-Hercosett treatment can cause wool to yellow. The dyeing effect is worse when the wool fiber turns yellower, especially for light colors. This phenomenon is to be avoided when using multiple padding enzyme treatment.

What is more, chlorination causes a large amount of chlorine to spread into the air. A strong pungent odor can be smelt during the chlorination process. This will seriously damage the health of the operator and destroy the atmosphere. AOX is also be generated in the process of chlorination which is difficult to decompose in wastewater [3]. In comparison, the multiple padding enzyme process has no AOX generation and the waste water is easy to biodegrade. In addition, the multiple padding enzyme process is a continuous process. It is not only eco-friendly but also highly efficient. This technology can replace the chlorine treatment of the wool shrinkproofing finishing.

## 4. Conclusions

To summarize, the multiple padding process can improve the efficiency of enzyme on wool fiber and reduce the enzyme treatment time. The area shrinkage of treated wool was 2.4% (lab) and 4.3% (plant) by the multiple padding process for 2.5 min. The tenacity loss of the treated wool was less in the plant (5.6%) than in the lab (7.4%). The adsorption performance of enzyme revealed that the multiple padding treatment can control the adsorbance of the enzyme onto the wool fiber and promote enzyme desorption and migration, so that the reaction between enzyme and wool fiber is controlled on the surface of the wool and the treatment is more uniform avoiding excessive damage to the wool fibers. Amino acid analysis and XPS analysis suggested that the multiple padding process obviously accelerated the damage of disulfide bonds. The removal efficiency of the disulfide bond was about 15 times as much as in the exhaustion treatment in 2.5 min. The reduction of disulfide bonds obviously promoted the hydrolysis of enzyme to the wool. The average catalytic rate of the padding process was 14 times faster than in the exhaustion process, and the process time (2.5min) decreased by 32.5 min compared with the exhaustion process (35 min). Due to the high efficiency of the enzyme and the short processing time, excessive damage caused by the extensive time enzyme exhaustion treatment to wool fibers can be avoided.

The multiple padding wool shrinkproofing process with the Savinase 16L and activator is continuous, easy to operate and has no AOX in the effluent. This technology was preliminarily industrialized at the Changshu Xinguang Wool Top Treatment Co. Ltd. (Changshu, China). It can be widely applied in industry and used in place of the environmental polluting chlorine treatment. AOX zero emission can be realized in wool shrinkproofing treatment in the near future. In addition, the results of this study are instructive in the research field of enzyme application.

## Figures and Tables

**Table 1:** Wool shrinkproofing properties and directional friction effect (D.F.E.).

Samples	Area Shrinkage/%	Static D.F.E./%	Kinetic D.F.E./%
Control	56.8 (0.53)	26.5 (0.17)	23.7 (0.43)
Exhaustion treatment	49.2 (0.67)	23.9 (0.33)	21.8 (0.62)
Padding treatment	2.4 (0.45)	9.3 (0.18)	8.1 (0.33)

Area shrinkage test was conducted after a 7A washing and consecutive 5A washing and flat-dry cycles 5 times (5 × 5A). The standard of machine washable is TM 31 5 × 5A area shrinkage ratio less than 8%.

**Table 2:** Fiber fineness and mechanical properties.

Samples		Fineness/μm	Strength/cN	Tenacity/×10^−2^cN/μm^2^	Retention Rate/%	
					Strength	Tenacity
Control		19.5 (0.21)	6.6 (1.01)	2.2	--	--
Exhaustion treatment		19.4 (0.43)	6.2 (1.57)	2.1	94.1	94.6
Padding treatment	1^#^	19.4(0.20)	6.5(1.21)	2.2	97.6	99.5
	2^#^	19.3(0.33)	6.3(0.85)	2.1	94.7	97.6
	3^#^	19.2(0.36)	6.1(1.10)	2.1	91.6	95.3
	4^#^	19.0(0.31)	5.9(0.83)	2.1	88.3	94.2
	5#	18.9 (0.20)	5.7 (0.93)	2.0	87.0	92.6

where 1^#^ means padding treated 1 time with 1 mL/L Savinase 16L and 2 mL/L activator, at 50 °C, pH 8.0,…, 5^#^ means padding treated 5 times with 1 mL/L Savinase 16L and 2 mL/L activator, at 50 °C, pH 8.0.

**Table 3:** Surface elements content of wool fiber after different processes.

Samples	Concentration of Elements (At%)			
	C1s	N1s	O1s	S2p
Control	75.9	7.8	11.6	3.2
Exhaustion treatment	77.2	9.6	11.4	1.9
Padding treatment	76.3	7.3	15.1	1.3

**Table 4:** Results of deconvolution analyses of S 2p peak for treated wool samples.

Samples	Relative Area of Different Chemical Bonds (%)	
	–S–S– (164.48 eV)	–SO_3_H (168.5 eV)
Control	100.0	<0.1
Exhaustion treatment	80.0	20.0
Padding treatment	19.6	80.4

**Table 5:** Color change after Chlorine-Hercosett treatment and multiple padding enzyme treatment.

Sample	L*	a*	b*	∆L*	∆a*	∆b*	CIE Whiteness
Control	87.9	−0.1	10.5	—	—	—	20.8
Chlorine-Hercosett treatment	90.7	−1.2	11.2	2.8	−1.1	0.7	24.6
Multiple padding enzyme treatment	90.4	−1.0	9.4	2.5	−0.9	−1.1	32.5
